# Modelling and synthesis of Magnéli Phases in ordered titanium oxide nanotubes with preserved morphology

**DOI:** 10.1038/s41598-020-64918-0

**Published:** 2020-05-15

**Authors:** Hammad Malik, Sayan Sarkar, Swomitra Mohanty, Krista Carlson

**Affiliations:** 1 Department of Materials Science and Engineering, University of Utah, Salt Lake City, Utah 84112 USA; 2 Department of Chemical Engineering, University of Utah, Salt Lake City, Utah 84112 USA

**Keywords:** Materials for devices, Materials for energy and catalysis, Nanoscale materials, Techniques and instrumentation, Theory and computation

## Abstract

The presence of Magnéli phases in titanium oxide nanotubes (NTs) can open up frontiers in many applications owing to their electrical and optical properties. Synthesis of NTs with Magnéli phases have posed a challenge due to the degradation and loss of morphology in NTs upon high-temperature treatments (>600 °C) in a reducing environment. This study reports on the synthesis of anodically formed NTs containing Magnéli phases through a double annealing route: oxygen (O_2_) annealing followed by annealing in 2% hydrogen with a nitrogen balance (2%H_2_-N_2_). The nucleation, growth, and transformation of anodized amorphous NTs into crystalline phases was investigated. The NTs obtained through this route were highly ordered and composed of mixed phases of anatase, rutile, and the Magnéli phase (Ti_4_O_7_). Experimental results from scanning electron microscopy (SEM), X-ray diffraction (XRD), scanning transmission electron microscopy (S/TEM), and Raman spectroscopy were combined with first principle calculations to develop an understanding of the sequential phase transformations during annealing. A predictive model was developed using density functional theory (DFT) to potentially predict the titanium oxides formed and their stability with reference to the mole fraction of oxygen. The change in the density of states (DOS), band structure, optical properties, and stability of phases are also discussed using DFT simulations. The combination of experimental characterization and modelling helped to understand the nucleation of anatase and rutile and the reorganization of these phases to form Magnéli phases on the anodized amorphous NTs through annealing treatment.

## Introduction

Magnéli phases are substoichiometric titanium oxide with chemical formula Ti_n_O_2n−1_ where n ranges from 4–9^1^. Magnéli phases are highly desirable because of their inertness in aggressive media^2^ and their conductance^3^; for example, at room temperature they are stable in 4 mol dm^−3^ sulfuric acid (H_2_SO_4_) with a projected half-life of 50 years^4^. Magnéli phases are semi-metallic in nature with electrical properties comparable to carbon^1,2^. There has been a significant amount of research on Magnéli materials due to the increasing demand for chemically inert, conductive materials^5^. Much of the research has been focused on the synthesis of Magnéli phase titanium oxide by reducing rutile under high temperatures 600–1000 °C^1^. Annealing in a reducing environment produces a number of defects in the TiO_2_ crystal structure, including oxygen vacancies (V_o_). The increasing number of V_o_ alters the Ti and O ratio, resulting in the formation of different suboxides (i.e., Magnéli compounds). Magnéli phases form from TiO_2_ according to the following reaction^6^:

1 $${{\rm{nTiO}}}_{2}+{{\rm{V}}}_{{\rm{O}}}\to {{\rm{Ti}}}_{{\rm{n}}}{{\rm{O}}}_{2{\rm{n}}-1}$$

These structural changes and oxygen deficiencies reduce the resistance of the oxides results in delocalized electrons that assist in electron pathway, thus increasing the conductivity^1,7^.

The formation of Magnéli phases by annealing in a 2% hydrogen gas with a nitrogen balance (2%H_2_-N_2_) is a sequential process; starting with anodized amorphous NTs, annealing initially nucleates anatase crystals that transform to rutile during high temperature treatment. After prolonged treatments in reducing environment, defects are introduced in rutile leading to thermodynamic instability and subsequent atomic reorganization into the stable Magnéli phases under the high temperature treatments. However, using this technique to form Magnéli phases within anodically formed NTs is challenging because of the structural changes encountered within the NTs at high temperatures. During annealing, the NTs are prone to atomic rearrangement with formation of V_o_ with concurrent morphology changes due to sintering, as nucleation and growth occurs within the NTs and at the NT-Ti substrate interface. These changes degrade the highly-ordered, thin walled (<10 nm) NTs until they are completely degraded onto the titanium substrate.

The present work introduces an annealing route that enables the formation of Magnéli phases while maintaining the morphology of the nanotubes. This double annealing route consist of oxygen (O_2_) annealing at 500 °C followed by annealing in a 2%H_2_-N_2_ environment at 600 °C. This route ensures the formation of Magnéli phases while preserving the NTs morphology. A predictive model was first developed using density functional theory simulations for predicting suboxide (Magnéli compound) formation at specific mole fractions of oxygen. Experimental investigations of the relationship between process and structure were then performed to validate the model.

## Results and Discussion

The annealing of as-anodized NTs causes the amorphous structure to undergo extensive changes that depend on the processing path and temperature. This link between processing and structure was used to successfully develop the double annealing route for obtaining NTs containing Magnéli suboxide, while preserving the nanotubular morphology. The key driving force behind the formation of the Magnéli phase was found to be the formation of oxygen vacancies (V_o_), which is also the major driver in the degradation of the NTs. In order to form Magnéli phases within NTs while maintaining their nanotubular structure, the changes in the morphology and crystalline phases with varying temperature and gaseous atmosphere (O_2_ and 2%H_2_-N_2_) was investigated. A successful route to obtain the desirable phase while maintain morphology was found by annealing in O_2_ at 500 °C with a subsequent anneal in 2%H_2_-N_2_ at 600 °C. Annealing in O_2_ enabled the nucleation of anatase and transformation of almost half of the nucleated anatase to rutile. Subsequent annealing in 2%H_2_-N_2_ at 600 °C then transforms some of the rutile into the Magnéli phases. Without the initial annealing in O_2_ (i.e., directly annealing in 2%H_2_-N_2_ from the amorphous state), the lack of oxygen creates so many V_o_ that the nanotubes begin to degrade, and the underlying titanium substrate over which the NTs are grown reduces and consumes the NTs.

### Density functional theory (DFT) for formation of magnéli phases

**Figure 1:**
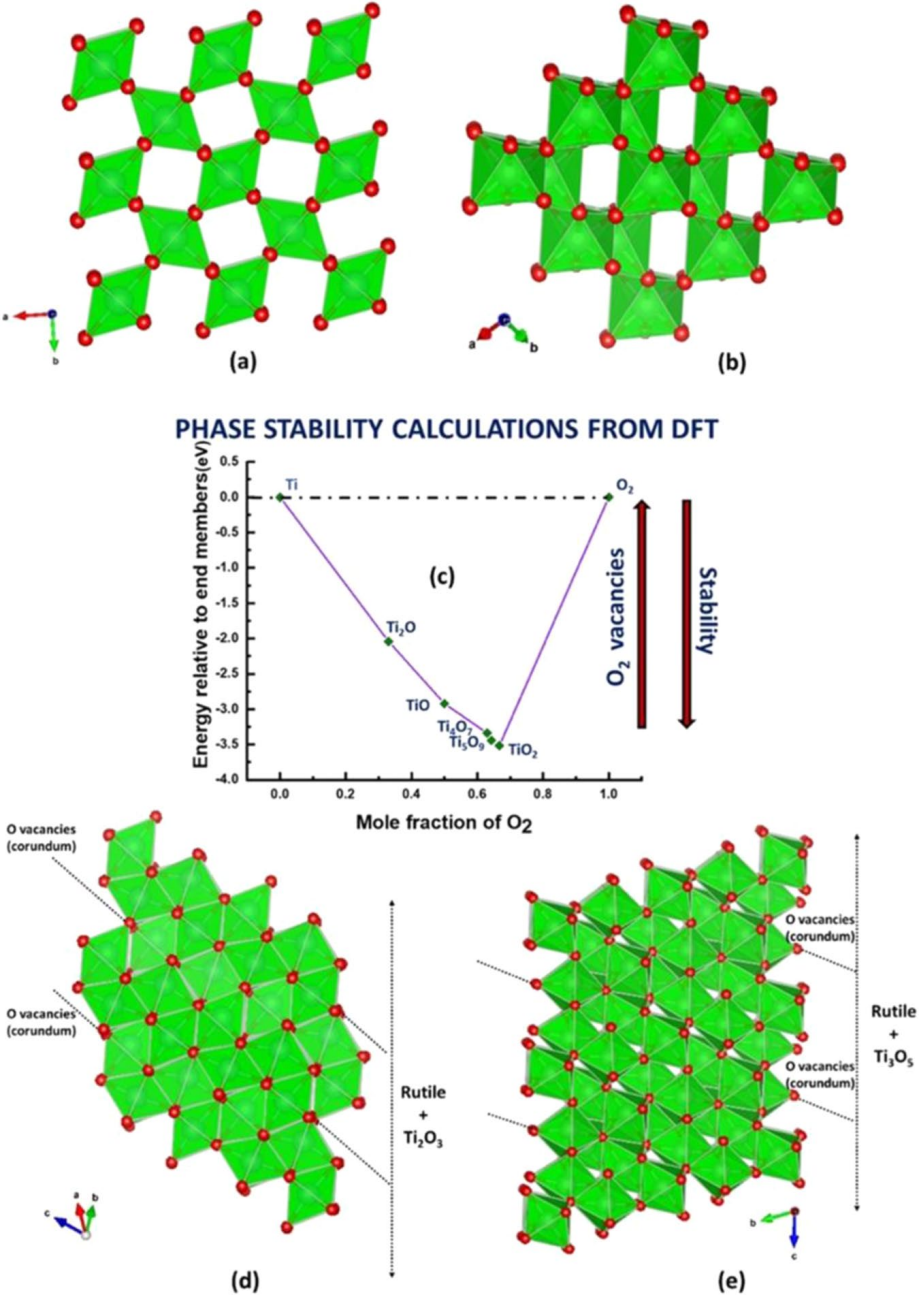
Crystal structure and relative stability of Magnéli phases. The 2 × 2 × 2 supercell (primitive unit cell) of tetragonal crystal (**a**) rutile and (**b**) anatase, and the triclinic crystal of the Magnéli phases (**d**) Ti_4_O_7_ and (**e**) Ti_5_O_9_. Ti atoms form a green octahedra surrounded by red coloured O atoms at the corners of the octahedra. The Magnéli phase Ti_4_O_7_ crystal system made of rutile chains along the c direction and Ti_2_O_3_ corundum bounded along the (001) planes (**d**). Likewise, the Magnéli phase Ti_5_O_9_ is composed of rutile chains and Ti_3_O_5_ corundum. Corundum in these Magnéli phases refer to shear planes which are induced due to depletion of O atoms. The relative stability of the Magnéli phases (**c**) in the TiO_2_ system for unit cell structures as calculated from DFT calculations. The stability diagram shows that Ti_5_O_9_ and Ti_4_O_7_ are the most stable Magnéli phases in the TiO_2_ system.

DFT was used to predict what TiO_2_ compounds would form based on the relative phase stability of unit cells of Magnéli phase in comparison to the stable TiO_2_ phase at ambient temperature. Figure 1c shows the DFT model that calculates and predicts thermodynamically stable Ti-O oxides by comparing the relative energies of different oxides to the end members Ti and O which was used as a reference for zero energy (0 eV). The model gives an inverse trend between the phase stability and the amount of oxygen vacancy V_o_ for the Ti-O oxides. From the first principle calculations, the formation energy of the unit cell was calculated based on the Linear Combination of Atomic Orbitals (LCAO) calculator^8,9^:

2 $${E}_{formationenergyofunitcell}=T[n]+{E}^{exc}[n]+{E}^{H}[n]++{E}^{ext}[n]-\sigma S$$

where

$$T[n]\equiv kinetic\,energy\,of\,the\,Kohn-Sham\,Orbitals,$$

$${E}^{exc}[n]\equiv exchange-correlation\,energy$$

$${E}^{H}[n]\equiv Hatree\,energy\,representing\,the\,electrostatic\,interaction$$

$$\sigma S\equiv entropy\,contribution$$

**Figure 2:**
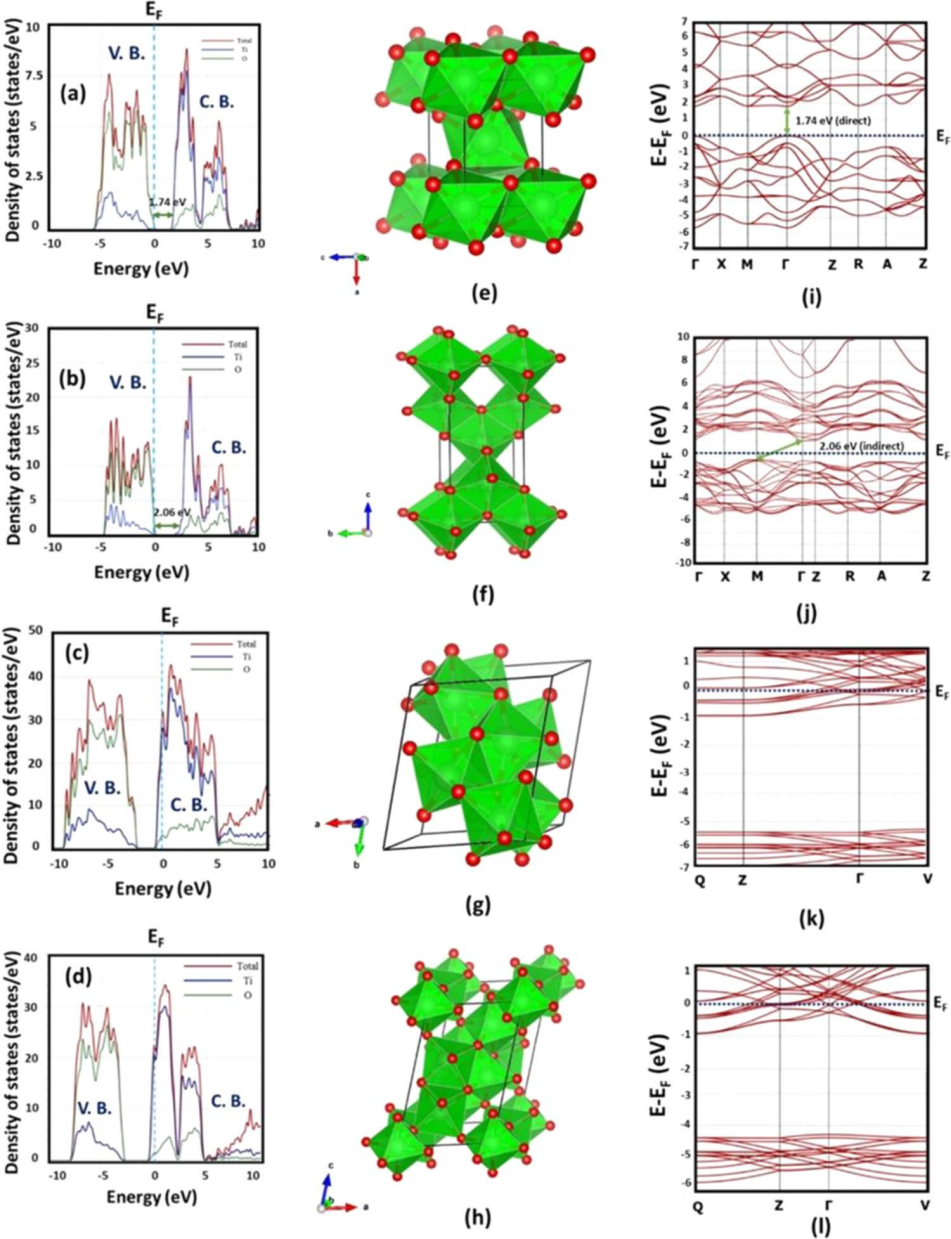
DFT based optimized unit cell, band structure and Density of States (DOS) diagram of TiO_2_ and Magnéli phases. The Normalized DOS diagram for (**a**) rutile (**b**) anatase (**c**) Ti_4_O_7_ (**d**) Ti_5_O_9_. Quantum Expresso based PBE optimization (without spin orbit coupling (SOC)) for unit cells of (**e**) rutile (**f**) anatase (**g**) Ti_4_O_7_ (**h**) Ti_5_O_9_. DFT based band structures of (**i**) rutile (**j**) anatase (**k**) Ti_4_O_7_ (l) Ti_5_O_9_. The band structures were calculated along the high symmetry directions of $\Gamma \to$ X $\to {\rm{M}}\to \Gamma \to {\rm{Z}}\to {\rm{R}}\to {\rm{A}}\to {\rm{Z}}$ for the tetragonal systems of rutile and anatase, symmetry direction of ${\rm{Q}}\to {\rm{Z}}\to \Gamma \to {\rm{V}}$ for triclinc systems of Magnéli phases of Ti_4_O_7_ and Ti_5_O_9_.

By dividing the total formation energy by the number of atoms in the unit cell to obtain the formation energy/atom, it was shown that the higher the formation energy/atom, the higher the stability of the structure. Figure 1c shows the relative stability of the non-stoichiometric Magnéli phases as a function of the mole fraction of O. The DFT calculation revealed that the most stable phase is TiO_2_ which showed a formation energy of −3.51 eV/atom. Amongst the sub oxides, the Magnéli phases of Ti_5_O_9_ and Ti_4_O_7_ were calculated to have highest stability of −3.44 eV and −3.42 eV, respectively. The Magnéli phases are derived from the natural occurring anatase and rutile (Fig. 1a,b)^10^. Ti_4_O_7_ (Fig. 1d) is comprised of periodic arrangement of rutile chains along the c-direction with a Ti_2_O_3_ structure along its boundary. The Ti_2_O_3_ corundum is made of TiO_6_ octahedral blocks that share faces. In this structure, the corundum bounds the rutile chains and represents the planes of crystallographic shear in Magnéli phase formed by the occurrence of O vacancies (i.e., the plane of defects)^11,12^. This Magnéli phase crystallizes in a triclinic structure with a *P* $\bar{1}$ space group. The Magnéli phase Ti_5_O_9_ has the subtle difference from the Ti_4_O_7_ in that the rutile planes are comprised of one extra plane of TiO_6_ atoms (Fig. 1e). The presence of V_o_ in the unit cell increases the volume of Ti_4_O_7_ (237.72 **Å**^3^) to Ti_5_O_9_ (302.38 **Å**^3^), which occurs when concentration of V_o_ formed becomes high enough to induce a shear operation in the rutile planes **(121)** $\frac{1}{2}$ **[0** $\bar{1}$ **1]**, resulting in subsequent displacements of atoms in the rutile plane^13,14^. This shear operation is associated with a dislocation in the direction [0 $\bar{1}$ 1] of the rutile structure along with shifting of the atoms along the c direction lying above the (121) plane. This dislocation increases the lattice constant c in these Magnéli phases as shown in Table 1^6^. Reduction in the Ti-O bond distances (4,6 pairs) from Ti_4_O_7_ (1.87** Å)** and Ti_5_O_9_ (1.86** Å)** respectively are observed (Fig. 2e–h). The difference between the DFT calculated unit cell lattice parameters and experimental values were within 6% of each other^15^.

**Table 1:** Lattice information for optimized unit cells and normalized fromation energy for TiO_2_ variants and other Magnéli phases.

Compound	Crystal Structure	Space-Group	Lattice-Parameters						Energy of formation/atom	Volume	Ti−O bond distance
			a (Å)	b(Å)	c (Å)	α	β	γ	eV	(Å^3^)	(Å)
Rutile	Tetragonal	*P4*_*2*_*/mnm* (136)	4.64	4.64	2.97	90°	90°	90°	−3.47	64.29	1.94
Anatase	Tetragonal	*I4*_*1*_*/amd* (No. 141)	3.8	3.8	9.74	90°	90°	90°	−3.51	140.95	1.94
Ti_4_O_7_	Triclinic	*P* $\bar{1}$(No. 2)	5.63	6.95	7.18	64.17°	71.10°	75. 11°	−3.42	237.72	1.87
Ti_5_O_9_	Triclinic	*P* $\bar{1}$(No. 2)	5.61	7.18	8.55	69.48°	75.19°	71.30°	−3.44	302.38	1.86

The normalized electron DOS for rutile, anatase, Ti_4_O_7_ and Ti_5_O_9_ are presented in Fig. 2. As shown in Fig. 2a and b for both rutile and anatase, the conduction band is mainly comprised of Ti 3d states, whereas the valence band from both is composed of O 2p electrons. However, in the case of Ti_4_O_7_ and Ti_5_O_9_, V_o_ tend to form an intermediate band comprised of pseudo-point defects^16^. This intermediate band forms in a reduced atmosphere when Ti^4+^ interstitials in the conduction band are converted into Ti^3+^, which are shallow donor states located below the conduction band minima (CBM). For Ti_4_O_7_ and Ti_5_O_9_, the intermediate band encompasses the zone of zero energy (Fermi level), thereby reducing the band gap to zero (i.e., semi-metallic).

The electronic DOS results are consistent with our band structure calculations (Fig. 2i–l). The band structure for rutile shows a direct band gap (1.74 eV) in the point of the Brillouin zone (Fig. 2i). However, in the case of anatase, the valence band minima (VBM) occurs at the M point of the Brillouin zone, whereas the conduction band maxima occur at the M point resulting in a non-direct band gap (2.06 eV). The DFT predicted band gaps of anatase and rutile were found to be lower as compared to their experimental band gaps of 3.2 and 3 eV respectively, which is expected since GGA-PBE exchange correlation used in our calculations underestimates the band gap^17^. For both Ti_4_O_7_ and Ti_5_O_9_, the intermediate band of pseudo defects (Ti^3+^) brings down the energy of the conduction band below the Fermi level (Fig. 2k,l). This induces semi-metallic states in the Magnéli phases in the Fermi level zone, making the electron behaviour similar to that of metals.

### Formation of Magnéli phases with in NTs

**Figure 3:**
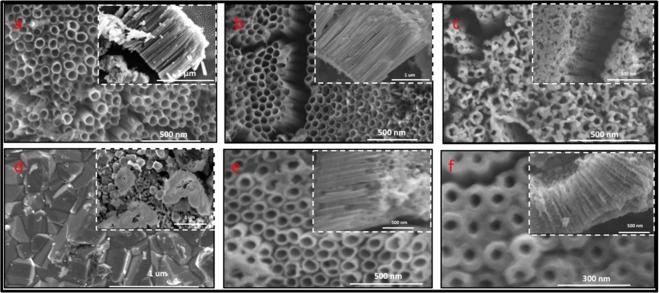
SEM images of TiO_2_ nanotubes. SEM images of nanotubes before and after annealing. (**a**) As anodized nanotubes (**b**)Nanotubes annealed in 2%H_2_-N_2_ at 600 °C, inset shows the nanotubes along the length. (**c**) Nanotubes annealed in 2%H_2_-N_2_ at 700 °C show degraded nanotubes (**d**) Nanotubes annealed in 2%H_2_-N_2_ at 800 °C show complete crystal structure. (**e**) Nanotubes annealed in O_2_ at 500 °C. (**f**) Nanotubes annealed in O_2_ at 500 °C and subsequent annealed in 2%H_2_-N_2_ at 600 °C.

**Figure 4:**
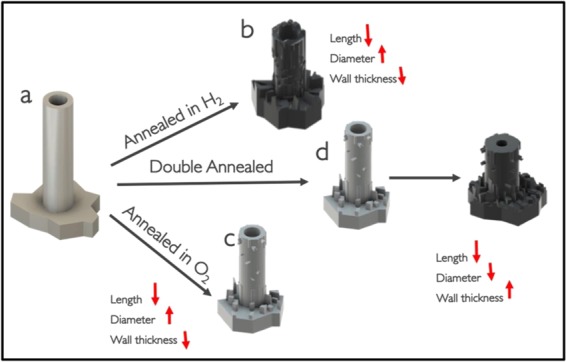
Schematic showing the morphological changes within NTs during annealing. (**a**) Anodized amorphous NTs (**b**) NTs annealed in 2%H_2_-N_2_ at 600 °C show degraded nanotubes with changes in color, and high density of crystal growth on the NT walls and the Titanium substrate carrying the NTs. (**c**) NTs annealed in O_2_ at 500 °C and subsequent annealed in 2%H_2_-N_2_ at 600 °C, stable NTs with increased wall thickness and decreased length, very high density of crystal growth and change in color. (**d**) NTs annealed in O_2_ at 500 °C showing stable NTs with comparatively smaller crystal growth structure.

NTs are desirable because of their ordered morphology and high surface area, however, if amorphous, their efficacy is limited by the small fraction of the charge carriers (i.e., electrons and holes). Amorphous NTs are considered electrical insulators at room temperature due to their low conduction and amorphous nature^18^. These traits make them unattractive for numerous applications where faster electron transfer is needed^19^. Annealing is performed to form a crystal structure within NTs with more desirable properties. Upon subjecting the anodized amorphous NTs (Fig. 4a) to direct annealing in an 2%H_2_-N_2_ environment, hydrogen interacts with the TiO_2_ lattice depending upon the operating temperature range^20^. At T < 300 °C, hydrogen physically interacts with the adsorbed oxygen of TiO_2_ lattice^20^. At T > 300 °C, electrons from hydrogen begin to transfer to the oxygen of TiO_2_ lattice leading to oxygen abstraction. This combination forms H_2_O that leaves the TiO_2_ surface, resulting in oxygen vacancies. At T > 450 °C, interactions between dissociated H and Ti^4+^ create Ti^3+^ interstitial defects^20^. During 2%H_2_-N_2_ annealing at T > 600 °C, the NTs begin to deform as the highly reducing environment deteriorates the outer surface whereas the bottom of the NTs begins to be consumed by the titanium oxide substrate (Fig. 4b), resulting in a large decrease in the wall thickness and the length of the NTs: The wall thickness decreases from 22 ± 3.47 nm for the as anodized NTs (Fig. 3a) to 14 ± 1.2 nm for 2%H_2_-N_2_ annealed, and the length decreases from 3 ± 0.33 μm to 1.7 ± 0.18 μm (Table 2). The decrease in the wall thickness is attributed to the loss of TiO_2_ lattice at the surface that are in direct contact with the reducing gas, whereas the decrease in length is due to the consumption of NTs by nucleation of denser crystals within the NTs and at the Ti-TiO_2_ interface. Additionally, the elevated temperature and inert atmosphere during annealing promote sintering^3^, which degrades the NTs morphology, resulting in a loss of ordered morphology and surface area.

**Table 2:** Dimensions of the amorphous as anodized nanotubes and nanotubes anneaeld at different temperatures and under different environment. Calculated for 10 nantubes for every sample.

TREATMENT	Length (μm)	Diameter (nm)	Wall thickness (nm)
Anodization	3 ± 0.33	57 ± 3.4	22 ± 3.47
2%H_2_-N_2_ at 600 °C	1.7 ± 0.18	67 ± 4.4	14 ± 1.2
O_2_ at 500 °C	1.2 ± 0.09	68 ± 2.5	16 ± 0.79
Double annealed at 600 °C	0.62 ± 0.31	50 ± 3.9	34 ± 3

The changes in morphology under reducing environment observed in this study are consistent with Sun *et al*.^21^. The nucleation and growth of crystals in NTs is a sequential process with anatase forming at T < 400 °C, followed by rutile at T > 400 °C. Once formed the rutile crystallites catalyses the anatase to rutile transformation and causes dimensional changes and deterioration of the NTs^22^. At even higher temperatures, and under reducing environment, the rutile crystals become highly defect laden and start reorganizing themselves to Magnéli phases. Annealing below T < 600 °C, the NTs only undergo major morphology changes resulting in shorter, thinner walled tubes (Fig. 3b). Above 600 °C, the tops of the tubes start to aggregate (Fig. 3c) and above 700 °C the NTs shrink and form a matted crystal surface resembling a granular structure over the substrate (Fig. 3d). Formation of Magnéli phases within the NTs through direct annealing cannot be attained and only possible by controlling the morphology with the crystal formation and crystal transformation process.

The fractions of anatase and rutile generated during the 2%H_2_-N_2_ and O_2_ annealing is important and this was using the Spurr equation^23^:

3 $${F}_{A}=100-\left(\frac{1}{1+0.8\left(\frac{{I}_{A}}{{I}_{R}}\right)}\right)100$$

where, F_A_ is a mass fraction of anatase, and I_A_ and I_R_ are the intensities of strongest anatase and rutile peaks, respectively. At 500 °C, 2%H_2_-N_2_ NTs were 90% anatase and 10% rutile. The growth of rutile crystals starts degrading the nanotubes by consuming the anatase crystals because rutile is denser than anatase and grows via surface diffusion from tube walls^24^.

The critical anatase to rutile conversion in the NTs is possible by one of the two routes. One is the conversion of anatase crystallites growing on the nanotube to rutile at elevated temperatures. The other route happens when rutile is nucleating at the Ti-NTs interface, nucleation is much faster at the interface due to the bulk of the material and subsequently these nucleated crystallites at the interface also nucleate and grow into the NTs^18^. During annealing NTs predominantly have the second route that is the nucleation and growth at Ti-NTs interface similar to previously reported by Zhu *et al*.^24^.

To form Magnéli phases within the NTs while preserving the morphology, the NTs were annealed in O_2_ at 500 °C with a subsequent annealing in 2%H_2_-N_2_ at 600 °C. This double annealing route was designed to create NTs that have rutile crystals (during O_2_ annealing), followed by the introduction of defects that led to the Magnéli phase formation (during 2%H_2_-N_2_ annealing). During O_2_ annealing, oxygen is retained in the titania lattice, which allows for more anatase to rutile conversion (1:1 ratio of rutile to anatase). This transformation preserves the NT morphology (Fig. 4d), in contrast to the defect formation that occurs during 2%H_2_-N_2_ annealing. However, the transformation to the denser rutile phase at the Ti-NT interface consumes the amorphous NTs, leading to a decrease in length to 1.2 μm (Fig. 3e). Subsequent annealing in 2%H_2_-N_2_ destabilizes the rutile structure as the hydrogen chemically interacts with the oxygen in the lattice to form H_2_O^20^. The destabilized rutile crystals transform to stable Magnéli phases. The competition between nanotube consumption and degradation causes a decrease in length to 0.62 ± 0.31 μm and an increase in wall thickness to 34 ± 3 nm (Fig. 3f). While this morphology change is significant (Fig. 4c), the double annealing route enables the creation of Magnéli phases in nanotubular ordered structures.

**Figure 5:**
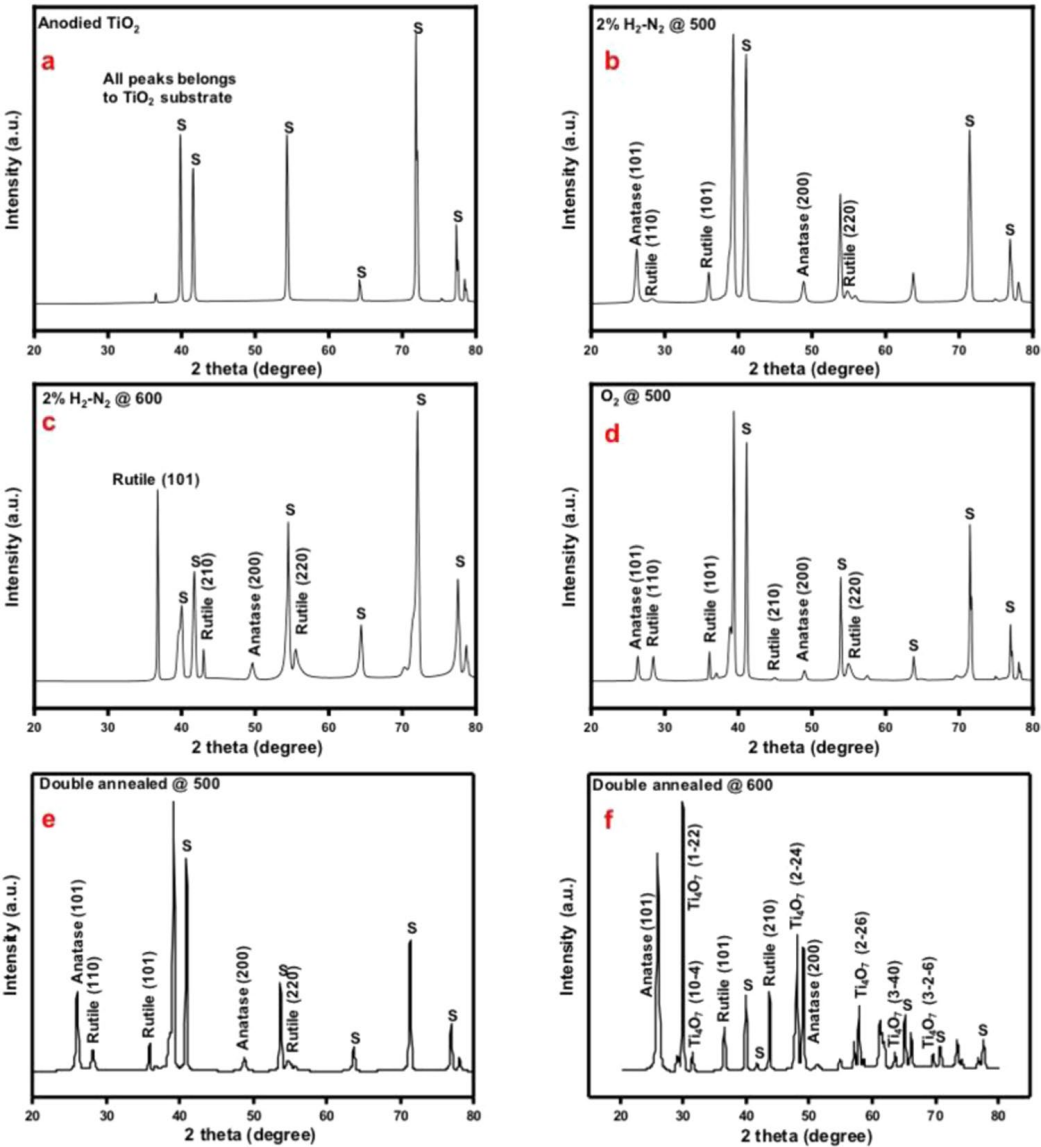
XRD patterns of TiO_2_ nanotubes annealed at different temperatures (°C). (**a**) Amorphous as anodized TiO_2_ nanotubes. (**b**) Patterns of nanotubes annealed in 2%H_2_-N_2_ at 500 °C show mixed anatase and rutile nanotubes with prominent peaks from titanium substrate (**c**) XRD pattern of nanotubes annealed in 2%H_2_-N_2_ at 600 °C with much prominent anatase and rutile peaks then titanium substrate peaks (**d**) XRD pattern of nanotubes annealed in O_2_ at 500 °C show similar pattern to 2%H_2_-N_2_ at 500 °C with mixed anatase and rutile nanotubes and prominent titanium substrate peaks (**e**) XRD pattern of nanotubes annealed in O_2_ at 500 °C and subsequent annealed in 2%H_2_-N_2_ at 500 °C show mixed anatase and rutile nanotubes 500 °C with prominent peaks for titanium substrate (**f**) XRD pattern of nanotubes annealed in O_2_ at 500 °C and subsequent annealed in 2%H_2_-N_2_ at 600 °C showing formation of Magnéli phase Ti_4_O_7_.

XRD patterns of the NTs were analysed to verify the crystal phase transformations with the different annealing treatments. The diffraction pattern of NTs before annealing showed peaks corresponding to the titanium substrate (Fig. 5a). The diffraction pattern of NTs annealed in 2%H_2_-N_2_ at 500 °C shows a mixture of anatase and rutile with almost 10% rutile (Fig. 5b). Annealing in this same environment at 600 °C, the concentration of rutile increases to approximately 92% (Fig. 5c). Annealing in 2%H_2_-N_2_ at T > 600 °C led to the rapid degradation of the nanotube morphology because of this anatase to rutile conversion, which is a reconstructive nucleation and growth process^25^. Although the anatase to rutile transformation at 600 °C is thermodynamically favourable, 100% rutile was not formed because the 3 hr annealing time is not long for the kinetics of the transformation as it decreases with the rise in temperature^26^.

NTs annealed in O_2_ at 500 °C show more rutile than those annealed in 2%H_2_-N_2_ (50% rutile compared to 10%, respectively) because in the absence of the chemical interactions between the NTs and hydrogen, nucleation and growth of rutile can easily proceed (Fig. 5d). Annealing NTs at 600 °C in O_2_ showed similar results (not shown). Only a slight broadening of the peaks was observed after a subsequent annealing of the O_2_ annealed NTs in 2%H_2_-N_2_ at 500 °C (Fig. 5e). The Magnéli phase Ti_4_O_7_ appears after the O_2_ annealed NTs are subsequent annealed in 2%H_2_-N_2_ at 600 °C (Fig. 5f). At this temperature, the rutile become defect laden and destabilized, which enables the reorganizational process where the rutile crystals undergo atomic rearrangements under oxygen depletion, forming Ti_4_O_7_^15^.

**Figure 6:**
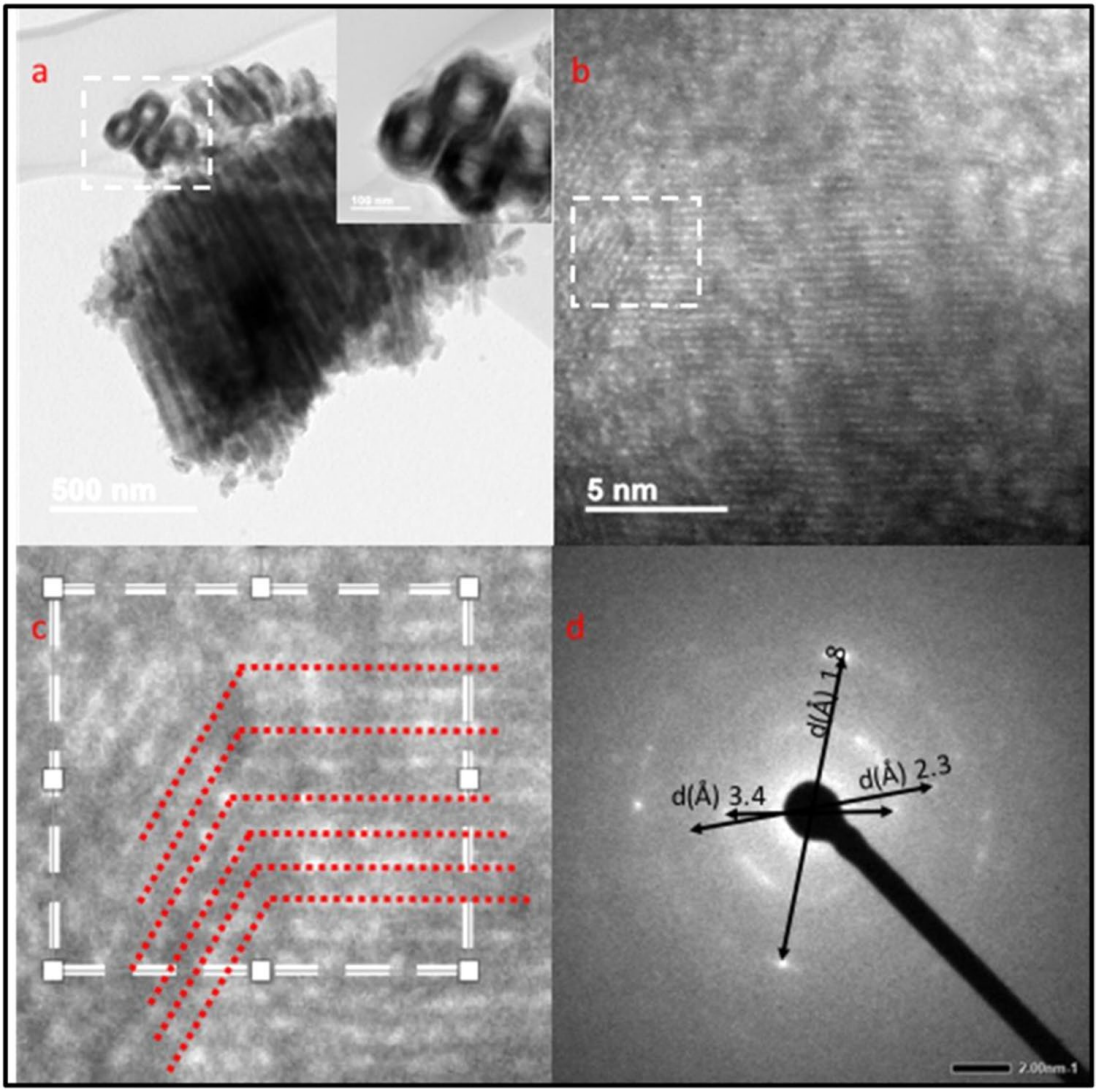
TEM images of double annealed at 600°C nanotubes. (**a,b**) TEM images of double annealed (O_2_ annealed at 500 °C followed by 2%H_2_-N_2_ at 600 °C) show the ordered NTs, (**c**) Magnified Magnéli twinned region; observation of Magnéli phase twinned regions (red dashed lines) on HR-TEM image (**d**) Selected area diffraction pattern showing presence of different crystal phases.

High-resolution transmission electron microscopy (HR-TEM) was used to confirm the formation of the Magnéli phase and to analyse the effects on the lattice during the double annealing route (O_2_ at 500 °C followed by 2%H_2_-N_2_ at 600 °C, Fig. 6). Figure 6b shows a well-defined lattice structure, indicating crystalline nanotubes. All Magnéli phases possess similar lattice parameters with the same triclinic crystal structure, which have overlying reflective peaks, making it difficult to distinguish between the phases^27^. Several locations on the HR-TEM image were used to calculate the lattice distance and the results are in good alignment with the XRD diffraction pattern of the same NTs. Lattice distances showed the samples to be a mixture of phases with characteristic anatase (3.5 Å for anatase [101]), rutile (3.2, 2.4 Å for rutile [110], [101]) and Ti_4_O_7_ (1.9,1.6 Å for Ti_4_O_7_ [2 $\bar{2}$ 4], [30 $\bar{4}$]).

The presence of twinned regions in the lattices of the HR-TEM images helped in developing an understanding of the formation mechanism of Magnéli phases (Fig. 6d). These twinned regions are formed by changes in crystals (anatase, rutile) due to reducing environment resulting in a high density of aperiodic stacking defects (crystallographic shear planes). These defects are ordered by long range ordered Magnéli phases forming the microtwinned Magnéli crystals^28^.

**Figure 7:**
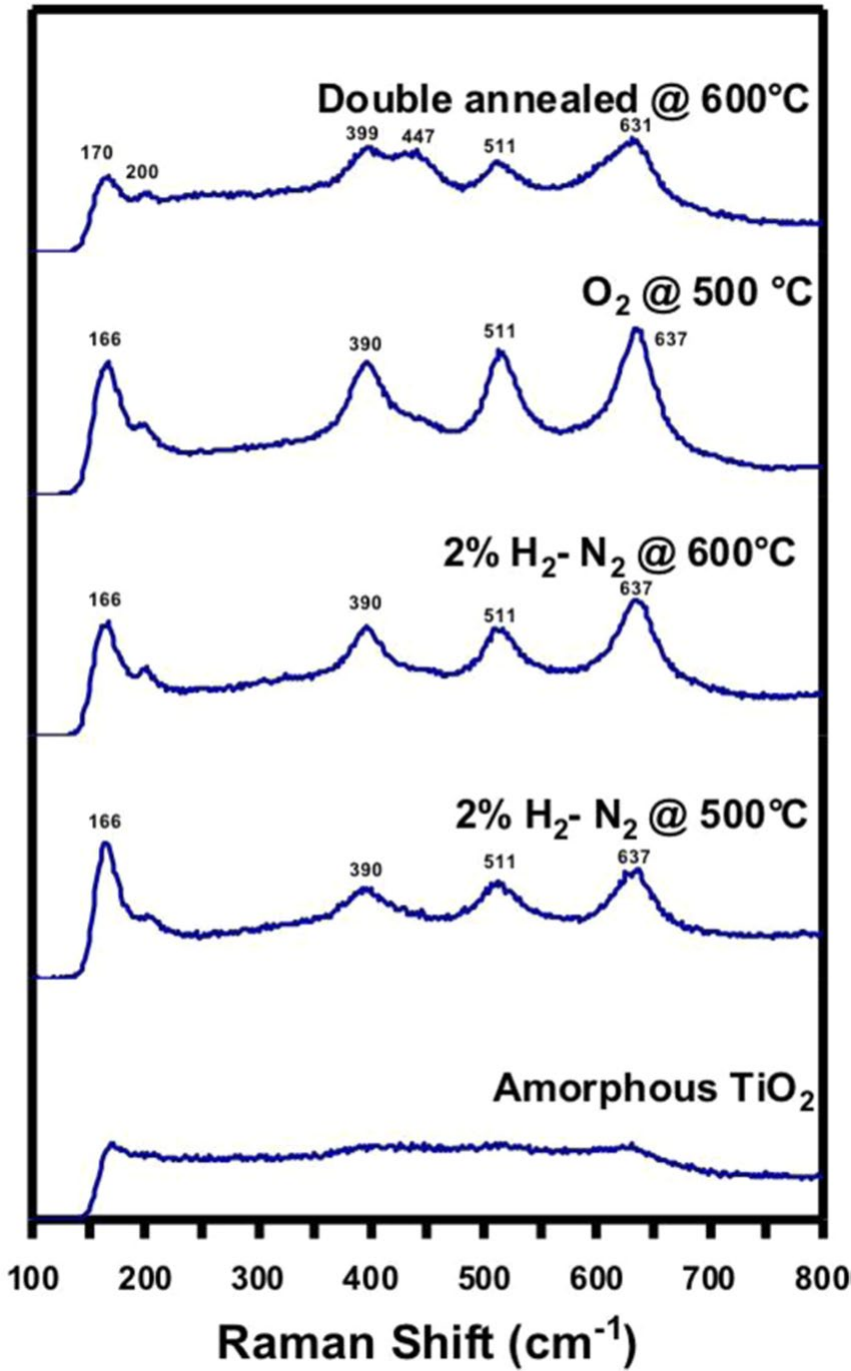
Raman Spectra of NTs. Raman spectra of amorphous NTs, 2%H_2_-N_2_ annealed at 500 °C, 2%H_2_-N_2_ annealed at 600 °C, O_2_ annealed at 500 °C, double annealed at 600 °C. Peak shifting with the peaks at 166 cm^−1^ and 390 cm^−1^ for 2%H_2_-N_2_ annealed at 500 °C, 2%H_2_-N_2_ annealed at 600 °C, O_2_ annealed at 500 °C samples blue shifted to 170 cm^−1^ and 399 cm^−1^ for double annealed NTs, due to a reduction in the Ti-O ratio causing formation of Magnéli phases. The reduction is further revealed by the oxygen deficiency weak broad peak at 200 cm^−1^. In double annealed NTs, appearance of the new peak at 447 cm^−1^ was observed indicative of the Magnéli phases.

Raman spectroscopy was used to examine the structural difference between the NTs annealed through different routes (Fig. 7). Raman is a useful tool for assessing the TiO_2_ stoichiometry because the spectra generated is solely from the oxygen stoichiometry^3^ and this changing stoichiometry assist in determining the phase changes. The decrease in Ti/O ratio causing the formation of Magnéli phases attributes to blue peak shifting around 400 cm^−1^ and appearance of the broad peak near the anatase peak at 151^3^. The anatase and the rutile are Raman active between 100–900 cm^-1^^29^ with tetragonal crystal structure belong to D $\frac{19}{4h}$ and D $\frac{14}{4h}$ space groups respectively^30^. The spectra for as anodized samples showed no crystallinity (Fig. 7). NTs annealed in 2%H_2_-N_2_ at 500 °C and 600 °C show similar spectra of a mixed phase with anatase and rutile bands. For the NTs annealed in 2%H_2_-N_2_ at 600 °C, the anatase peaks at 166 cm^−1^ decreased while the rutile peaks intensified. This peak intensification for 2%H_2_-N_2_ annealed at 600 °C is a result of the higher fractions of rutile, confirming the XRD results. The spectra of NTs annealed in O_2_ at 500 °C had the same peak positions as the 2%H_2_-N_2_ annealed NTs but intensified peaks, attributed to oxide layer formation on the substrate. Peak shifting, appearance of weak broad peaks and completely new peaks due to formation of Magnéli phases in double annealed NTs are in alignment to previously reported by other researchers^3^. For the double annealed NTs, the peaks at 166 cm^−1^ and 390 cm^−1^ are blue shifted to 170 cm^−1^ and 399 cm^−1^, respectively. These peak shifts are due to a reduction in the Ti/O ratio^3^. The oxygen deficiency is further revealed by a weak broad peak at 200 cm^−1^. The formation of defects and reduction of Ti/O ratio are due to formation of Magnéli phases. In these NTs, the anatase peak intensity at 170 cm^−1^ is decreased and the appearance of the new peak at 447 cm^−1^ is indicative of Magnéli phases.

## Conclusion

This study introduced a novel double annealing route (O_2_ annealing at 500 °C followed by 2%H_2_-N_2_ annealing at 600 °C) for the formation of the Magnéli phases within the anodized NTs while preserving their highly ordered morphology. A model was developed using DFT for predicting the stability of titanium oxide phases formed with reference to the mole fraction of oxygen. DFT simulations were also used to estimate the density of states (DOS), band structure, and optical properties of the potential oxides formed. The characterization helped in understanding the morphological, structural, and dimensional changes occurring in the NTs during annealing. The benefits and limitations of annealing the anodized NTs in reducing and oxidizing conditions were analysed. These findings assisted in controlling the process-structure relationship and in developing a synthesis route that allows for the formation of mixed phase NTs with desirable Magnéli phases (Ti_4_O_7_) while maintaining their highly ordered morphology.

## Materials and Methods

### Synthesis of titanium nanotubes

Titanium oxide nanotubes were synthesized according to the electrochemical anodization method from previous work^31–33^. A 99.99% titanium foil (CAS Number 7440-32-6, Alfa Aesar) was used as the starting material for growing NTs. The titanium foil was cut into 2.2 × 2.2 cm strip. Prior to electropolishing and anodization, these foils were ultrasonicated in 15 vol% Hydrochloric acid (CAS Number: 7647-01-0, Sigma-Aldrich) and deionized water (DI). The cleaning and drying was followed by electropolishing. Electropolishing and anodization were carried out at room temperature in a two-electrode electrochemical cell. Titanium foil was used as an anode and platinum gauze was used as a cathode. The electropolishing solution was vinegar with 5% acidity (Kroger white vinegar). During electropolishing the cell was held at 25 volts for 1 minute. The electropolished foils were ultrasonicated in ethanol and DI-water to remove any organic contaminants before subjecting to anodization. Self-aligned nanotubes were grown by anodizing polished foils in an acidic ethylene glycol solution with 96.5 wt% ethylene glycol (CAS Number: 107-21-1, Fisher-Scientific), 0.5 wt% ammonium fluoride (CAS Number: 12125-01-8, Sigma-Aldrich) and 3 wt% DI. For anodization the cell was held at 30 volts for 60 minutes to form NTs. After anodization, the foils with NTs were ultrasonicated in deionized water and air dried overnight^32^. To improve the properties of these NTs, the amorphous as anodized NTs were subjected to annealing to become crystalline. Direct annealing of the anodized NTs was done in Oxygen (O_2_) at 500 °C or ultra-pure 2% hydrogen with a nitrogen balance (2%H_2_-N_2_) 500 °C. Some of the anodized NTs were subjected to double annealing route, where the NTs were O_2_ annealing at 500 °C followed by 2%H_2_-N_2_ annealing at 600 °C. The annealing furnace was set to reach the desired temperature in 60 minutes and retain the temperature for 180 minutes finally cooling back to room temperature in 60 minutes.

### Characterization of nanotubes

Morphology of amorphous and annealed NTs was observed using a field emission scanning electron microscope (FE-SEM, Hitachi, S-4800). The dimensions (Length, wall thickness, diameter) and uniformity of NTs were analysed using ImageJ software. A total of 10 nanotube dimensions for each sample was measured and the standard deviation for each dimension was also calculated. The crystal phases present within the NTs after annealing were observed using x-ray diffractometer (Rigaku MiniFlex 600) with CuKα radiation (λ = 1.54 A) between 2θ = 20°–80° at a rate of 0.025°/s. The XRD pattern were analysed using PDXL software. The Fast Fourier Transform (FFT) and Selected Area electron Diffraction (SAED) patterns were generated using high resolution transmission electron microscope (S/TEM, JEOL JEM-2800), and were analysed using Gatan, and ImageJ. Raman spectra were produced using micro-Raman spectroscopy (WiTec AlphaSNOM).

### DFT modelling

The band structures were calculated along the high symmetry directions of $\Gamma \,\to$ X $\to {\rm{M}}\,\to \Gamma \to {\rm{Z}}\to {\rm{R}}\to {\rm{A}}\to {\rm{Z}}$ for the tetragonal systems of rutile and anatase, symmetry direction of ${\rm{Q}}\to {\rm{Z}}\to \Gamma \to {\rm{V}}$ for triclinc systems of Magnéli phases of Ti_4_O_7_ and Ti_5_O_9_. Norm-conserving pseudopotential was used under the framework of General Gradient Approximation (GGA) along with the implementation of Perdew-Burke-Ernzerhof (PBE) exchange-correlation functional for density functional theory calculations^16^. The plane wave pseudopotential was used within the QUANTUM-ESPRESSO^22^ package with the aid of BURAI 1.3 as a GUI for the first principle calculations of TiO_2_ and other Magnéli variants. For each of these compounds, unit cells were used for structural optimizations during which limited memory Broyden-Fletcher-Goldfarb-Shanno (LBFGS) algorithm was used and the formation energy per atom total energy was calculated by using PBE exchange-correlation functional^34^. During the structural cells of TiO_2_ and other Magnéli variants was then used for band structure and Density of States calculations, keeping the electronic occupation fixed as in case of semiconductors. A dense (5 × 5 × 5) Monkhorst-Pack k-point sampling was used for Brillouin-zone integration. The kinetic energy cut-off for the wave function was 340 Ry and the corresponding value for charge density was 3400 Ry.

## Data Availability

Data available upon request from Krista Carlson (krista.carlson@utah.edu).
